# A Beta-splitting model for evolutionary trees

**DOI:** 10.1098/rsos.160016

**Published:** 2016-05-11

**Authors:** Raazesh Sainudiin, Amandine Véber

**Affiliations:** 1 School of Mathematics and Statistics, University of Canterbury, Private Bag 4800, Christchurch 8041, New Zealand; 2 CMAP-CNRS, Ecole Polytechnique, 91128 Palaiseau Cedex, France

**Keywords:** random evolutionary trees, Beta-splitting model(s), speciation and extinction model, binary search trees

## Abstract

In this article, we construct a generalization of the Blum–François Beta-splitting model for evolutionary trees, which was itself inspired by Aldous' Beta-splitting model on cladograms. The novelty of our approach allows for asymmetric shares of diversification rates (or diversification ‘potential’) between two sister species in an evolutionarily interpretable manner, as well as the addition of extinction to the model in a natural way. We describe the incremental evolutionary construction of a tree with *n* leaves by splitting or freezing extant lineages through the generating, organizing and deleting processes. We then give the probability of any (binary rooted) tree under this model with no extinction, at several resolutions: *ranked planar trees* giving asymmetric roles to the first and second offspring species of a given species and keeping track of the order of the speciation events occurring during the creation of the tree, *unranked planar trees*, *ranked non-planar trees* and finally (*unranked non-planar*) *trees*. We also describe a continuous-time equivalent of the generating, organizing and deleting processes where tree topology and branch lengths are jointly modelled and provide code in SageMath/Python for these algorithms.

## 1. Introduction

In the last couple of decades, many models of random evolutionary trees have been introduced and studied, as reviewed by Mooers & Heard [1] and Morlon [2]. Most of them are formulated in terms of (constant or variable) individual species diversification rates mirroring the influence of particular features such as species age, trait, available niche space, etc. In this way, they propose an evolutionary explanation for the shapes and branch lengths observed in some reconstructed real trees. Many of these models cannot jointly model the branch lengths and the tree topologies or shapes, are quite complex to analyse and have limited identifiability [2]. Note that, although we adopt here the terminology of evolutionary biology, the same kind of questions appear in other domains such as developmental biology (with cell lineage diagrams, cf. [1, p. 48]) or epidemiology [3]. The model developed in this paper may thus be of interest in these other contexts.

Even though the branch lengths of a phylogenetic tree give potentially precise indications on the individual diversification rates, their estimations may be subject to appreciable errors due to the difficulty of their reconstruction. On the other hand, the tree topology has a discrete nature that is somewhat easier to handle (for computation or comparison purposes, for example), and it already brings a lot of information on the phenomena shaping the clade diversity [1]. In particular, many works focus on the balance of a tree, measured by a diverse class of indices (e.g. Colless index, cf. [4] and Sackin index, cf. [5]). Of course many diversification mechanisms can lead to the same phylogenetic tree balance [6] and so such indices cannot be used on their own to characterize the way the reconstructed tree was generated. However, they may be used to rule out some scenarii. For example, several papers [1,7,8] point out the fact that the reconstructed trees of the TreeBase database are on average much more unbalanced than expected under the most well-known model of speciation, the *Yule model* [9]. In this model, every species branches into two species at the same rate (which may vary in time but remains identical for all species), and there is no extinction. The Yule model is the best known example of an evolutionarily interpretable model of speciation due to the following three features:

— it is based on an incremental evolutionary construction whereby the tree grows by splitting one of the current leaf nodes which represent the set of extant lineages,
— it can be defined jointly on the product space of tree topologies and branch lengths, and
— the distribution it induces on coarser resolutions of the tree space can be obtained.

Several models introduced in the literature are not evolutionarily interpretable in the above sense. The main objective of this paper is to formulate an evolutionarily interpretable parametric family of models that includes the Yule model as well as many others in the literature that originally lacked evolutionary interpretability.

Aldous [10] introduces a one-parameter family of random cladograms, called the *Beta-splitting model*. Here a cladogram is defined as a binary tree shape with a specified number of tips (or leaves) in which there is no ‘left’ and ‘right’ ordering of the child nodes of an internal node (in other words, the tree is *non-planar* and *unranked* as defined below). The leaves are labelled by the sampled species, or by {1,…,n} for simplicity. The parameter β>−2 modulates the shape and balance of the tree produced by this model by determining the split distribution of a node subtending *m* leaves. More precisely, Aldous' recursive construction involves a fixed *n*, the number of leaf nodes representing the extant species in a tree with at least two leaves and $\left\{q_{n}^{\beta }(i):1,2,\ldots ,n-1\right\}$, a symmetric probability distribution (i.e. $q_{n}^{\beta }(i)=q_{n}^{\beta }(n-i)$) which specifies the numbers *i* and n−i of descendants along the two branches emanating from the root node of the tree. Once this split (i,n−i) is fixed, the construction carries on recursively in the two subtrees pending from the root, with respective numbers of leaf nodes *i* and n−i, and stops when all subtrees considered have only one leaf. In the Beta-splitting model with β>−2, the split distribution $q_{n}^{\beta }$ takes the form

1.1 $$q_{n}^{\beta }(i)=\frac{1}{a_{n}}\left(\begin{array}{c} n\\ i \end{array}\right)\int_{0}^{1}x^{i+\beta }{\left(1-x\right)}^{n-i+\beta }\;dx$$

for 1≤i≤n−1, where $a_{n}$ is a normalizing factor given by

$$a_{n}=\int_{0}^{1}(1-x^{n}-{\left(1-x\right)}^{n})x^{\beta }{\left(1-x\right)}^{\beta }\;dx.$$

This *Markov branching model* has now become a reference in the literature [6,8,11], in particular, because it provides a family of random tree topologies indexed by a single parameter, which contains the most commonly used Yule tree (β=0) and proportional to distinguishable arrangements (or PDA) model in which every cladogram is equally likely ($\beta =-\frac{3}{2}$). The parameter *β* tunes the balance of the tree, since ‘β=−2’ corresponds to the totally unbalanced tree or comb, whereas the generated trees become more and more balanced as *β* tends to infinity. Aldous [7] also proposes a measure of the balance of a tree which has the advantage of being independent of the tree size, at least for large *n*'s: the median of the split distribution $q_{n}^{\beta }$. This measure is used to perform maximum-likelihood estimation of *β* or to compare the global balance of several trees [7,8].

Unfortunately, Aldous, being unable to find an appropriate underlying process (cf. [10, Section 4.3]) in his own words, ‘resort(s) to pulling a model out of thin air’ [10, Section 3]. Since the number of leaf nodes has to be known before recursive splitting begins, Aldous' Beta-splitting model is not based on an incremental evolutionary construction or defined jointly on the product space of tree topologies and branch lengths for every value of β>−2, and thus lacks evolutionary interpretability in our sense.

Subsequently, several other families of random tree topologies have been introduced, in particular Ford's alpha-model [12] in which branches are added one after another to the tree until it has the desired number of leaves. The parameter α∈[0,1] there serves to give a weight to each existing edge in the tree and then choose which one will be split to insert the next edge. Ford's alpha-model also lacks evolutionary interpretability since new species can arise not just from the currently extant leaf lineages but from any ancestral lineage that is currently extinct. Blum & François [8] introduced an evolutionary Beta-splitting model based on ideas of Kirkpatrick & Slatkin [13], and Aldous [10]. The idea is that the ‘speciation potential’ is shared between the two offspring species in a random way, as may occur, for example, in the cases where speciation is influenced by available niche or geographical space that is shared between the two new species. In this model, a (rooted binary non-planar) tree is constructed incrementally by starting from a single node (the root) with speciation rate (or ‘potential’) 1. When this first species branches, a parameter $p_{1}$ is sampled in [0,1] according to a Beta(β+1,β+1) distribution (the definition of the Beta distribution is recalled below). Then the first offspring species is given the speciation rate $p_{1}$, and the second the speciation rate $1-p_{1}$. The next species to split is thus the first one with probability $p_{1}$, or the second one with probability $1-p_{1}$. Carrying on the construction, upon the split of a species with speciation rate *λ*, a new parameter $p_{i}$ is sampled independently of the previous ones according to the same Beta(β+1,β+1) distribution, and the two sister species receive the speciation rates $\lambda p_{i}$ and $\lambda (1-p_{i})$. Then, each species is the next one to branch with a probability equal to its speciation rate/potential.

Though the Blum–François and the Aldous Beta-splitting models coincide for β=0, in general they do not yield the same distribution on cladograms. See the supplementary material of Blum & François [8] for a discussion of the relationships between the two families of processes. Nevertheless, the principles behind the two constructions are similar and the Blum–François model offers an approximate evolutionary construction of Aldous' Beta-splitting model, with a slightly restricted range of parameters (β>−1 instead of β>−2). Below, we argue that the range of topologies covered by the Blum–François model is quite wide as well, since ‘β=−1’ corresponds to the totally unbalanced trees while ‘β=∞’ corresponds to highly balanced trees. For the reasons expounded in this paragraph, we feel that this model has not yet received the attention it deserves in the phylogenetics community (or other communities as explained earlier), in particular because it is only sketchily described in Blum & François [8].

In this article, we extend the Blum–François model by allowing asymmetric Beta-distributions for the split distribution. That is, the fraction of ‘speciation potential’ allocated to the first offspring species is now distributed according to a Beta(α+1,β+1) distribution, for some α>−1 and β>−1. Of course this lack of symmetry makes sense only if we distinguish a first and second (or later ‘left’ and ‘right’) offspring species. This distinction appears naturally when we think of speciation as being the creation of a new species and the continuation of the mother species, in which case the two species will not play a symmetric role and have *a priori* no reason to speciate at the same rate (e.g. [14]). In the context of transmission trees in epidemiology [15], the left branch keeps track of the infector and its ‘infection potential’, while the right branch keeps track of the infectee and its ‘infection potential’ for each infection event recorded by the internal branch. The same distinction is true of cell lineage diagrams where the left branch can track the sister cell upon division using some measurable feature such as having more DNA damage than the sister cell along the right branch [16].

In order to formalize more precisely how these Beta-splits create a given topology of interest, we consider four types of (rooted binary) trees:

— *Ranked planar trees*: In this case, we distinguish the left and right child nodes of an internal node, and every internal node is labelled by an integer keeping track of the ordering in which the splits occur during the construction of the tree. Since a binary tree with *n* leaves has n−1 internal nodes, the labels thus run from 1 (the root) to n−1 (the last split).
— *Unranked planar trees*: Left and right child nodes are distinguished, but the internal nodes are not labelled (so that the order of the splits is not recorded).
— *Ranked non-planar trees*: In this case, the internal nodes are ranked and labelled according to the splitting order, but left and right child nodes play equivalent roles.
— *Trees*: Unranked and non-planar trees. Aldous' cladograms are such trees whose leaves are further labelled by the *n* taxa.

Indeed, as explained above, planarity can be interesting when the two sister species do not necessarily evolve according to the same mechanisms. The ranking of the internal nodes is a way to include some information on relative speciation times without keeping track of the full set of speciation times (e.g. [17]). Furthermore, various tree shape statistics are functions of the unranked non-planar trees or cladograms without leaf labels. Explicit expressions for the probability of any tree at each of these four resolutions are not available in the literature for the Beta-splitting models of Aldous or Blum–François. Thus, another contribution of this paper is the set of explicit expressions for the probability of any tree at the resolutions of ranked planar and unranked planar trees for any *α* and *β* and for the probability of any tree at the resolutions of ranked non-planar and unranked non-planar trees for any α=β.

We first focus on the finest of these four tree resolutions, that of ranked planar trees. We introduce the generalization of the Blum–François Beta-splitting model by decomposing the construction of a random ranked planar tree with *n* leaves into two steps. First, we sample a *generating sequence* ${\left(G_{i}\right)}_{i\geq 1}$, which is a realization of a sequence of independent and identically distributed random variables which, at each step *i*, will determine the choice of the next leaf to be split and the fraction of ‘speciation potential’ allocated to the left child of that former leaf. Once this generating sequence is fixed, we define a (non-random) *organizing process* that turns the generating sequence into a ranked planar binary tree with the desired number of leaves. Each of these leaves is labelled by a subinterval of [0,1] whose length is the speciation potential of the corresponding species. The intervals take part in the choice of the next leaf to be split. This construction enables us to add species extinction by a similar mechanism, thanks to a *deleting process* that encodes the freezing of some leaf nodes with a given probability δ∈[0,1). A frozen leaf can no longer evolve, and thus represents a species which is either extinct or no longer able to diversify. See the next section for a precise description of the generating, organizing and deleting processes.

Our next task is to describe the distribution on ranked planar trees corresponding to a given pair of parameters α,β>−1, as well as the distribution on the three coarser tree resolutions induced by this construction. We provide several examples in the case α=β of Blum & François [8], in particular to discuss the balance of the trees obtained as a function of *β*. For completeness, we also propose a continuous-time process of leaf splitting and freezing such that the shape of the tree obtained after *N* events (regardless of branch lengths) has the same distribution as that obtained through the generating, organizing and deleting processes after *N* steps. Finally, in the electronic supplementary material, we give SageMath/Python code to produce these trees at several resolutions as well as a demonstration of the code for the case of the Yule process with four leaves.

In the electronic supplementary material, we also discuss a reversibility result describing how to choose a pair of sibling leaves (a *cherry*) in an unranked planar tree with n+1 leaves created through the generating and organizing processes with *n* steps, in such a way that the tree with *n* leaves obtained by removing this cherry has the same law as a tree we would have obtained from the *GO* processes with only n−1 steps. This last result is on *sampling consistency* of our evolutionarily interpretable Beta-splitting model, which unlike Aldous' model (cf. [10, Section 6.3]), does not naively satisfy equivalence in distribution between (i) constructing a tree with n+1 leaves and then removing one leaf at random and (ii) constructing a tree directly with *n* leaves. Our result shows that in the unranked case (planar or non-planar), there is a natural but non-uniform way of choosing a terminal split to remove to obtain a tree with the same distribution as if it had been produced directly with the reduced number of leaves.

Let us end this section with some notation. To match the standard definition of the Beta distribution, for any α,β>0 we call B(α,β) the distribution on [0,1] with density $B{\left(\alpha ,\beta \right)}^{-1}x^{\alpha -1}{\left(1-x\right)}^{\beta -1}$, where

1.2 $$B(\alpha ,\beta ):=\int_{0}^{1}x^{\alpha -1}{\left(1-x\right)}^{\beta -1}\;dx.$$

If α=β, this distribution is symmetric: if X∼B(β,β), then 1−X∼B(β,β).

In all that follows, we shall consider the B(α+1,β+1) distribution (for α,β>−1), with density proportional to $x^{\alpha }{\left(1-x\right)}^{\beta }$. This choice corresponds to the density used in the Aldous and Blum–François Beta-splitting models in the symmetric case α=β.

## 2. An evolutionary construction

We fix α,β>−1.

### 2.1. The generating sequence

Let $\left(B_{1},B_{2},\ldots \right)$ be a sequence of independent and identically distributed (i.i.d.) random variables, with the B(α+1,β+1) distribution. Let also $\left(U_{1},U_{2},\ldots \right)$ be a sequence of i.i.d. random variables with the uniform distribution on [0,1], that is independent of $\left(B_{1},B_{2},\ldots \right)$. Thus, each of these variables takes its values in [0,1]. We call ${\left(G_{i}=(U_{i},B_{i})\right)}_{i\in N}$ the *generating sequence*. It will be the basis of an incremental construction of a ranked planar binary tree with *n* leaves and n−1 internal nodes.

Remark 2.1 Here we use the B(α+1,β+1) distribution, because it gives us a two-parameter family with a wide range of possible behaviours for the corresponding trees (as we shall see later). In general, we may take ${\left(B_{i}\right)}_{i\in N}$ to be a sequence of i.i.d. variables with some common distribution *F* on [0,1]. Even more generally, we may take a sequence with an arbitrary dependence of each $G_{i}$ on the previous values $\left(G_{1},\ldots ,G_{i-1}\right)$.

### 2.2. The organizing map

Let us now describe the deterministic mapping that takes a realization of the generating sequence ${\left(G_{i}\right)}_{i\in N}$ and turns it into a planar binary tree in which the internal nodes are labelled by an integer and the leaves are labelled by a subinterval of [0,1]. As we shall see below, the integer labels of the internal nodes will give the order in which these nodes have been split during the construction. The interval labels of the leaves will form a partition of the interval [0,1] and will be used to decide which leaf is split and becomes an internal node in the next step.

Let ${\left(g_{i}=(u_{i},b_{i})\right)}_{i\in N}$ be a realization of the generating sequence. The organizing map O(g) proceeds incrementally as follows, until the tree created has *n* leaves. We start with a single root node, labelled by the interval [0,1].

— Step 1: Split the root into a left leaf labelled by $\left[0,b_{1}\right]$ and a right leaf labelled by $\left[b_{1},1\right]$. Change the label of the root to the integer 1.
— Step 2: If $u_{2}\in [0,b_{1}]$, split the left child node of the root into a left leaf and a right leaf, respectively, labelled by $\left[0,b_{1}b_{2}\right]$ and $\left[b_{1}b_{2},b_{1}\right]$. If $u_{2}\in [b_{1},1]$, then instead split the right child node of the root into left and right leaves with respective labels $\left[b_{1},b_{1}+(1-b_{1})b_{2}\right]$, $\left[b_{1}+(1-b_{1})b_{2},1\right]$. Label the former leaf that is split during this step by 2.
— Step *i*: Find the leaf whose interval label [a,b] contains $u_{i}$. Change its label to the integer *i* and split it into a left leaf with label $\left[a,a+(b-a)b_{i}\right]$ and a right leaf with label $\left[a+(b-a)b_{i},b\right]$.
— Stop at the end of Step n−1.

**Figure 1.**
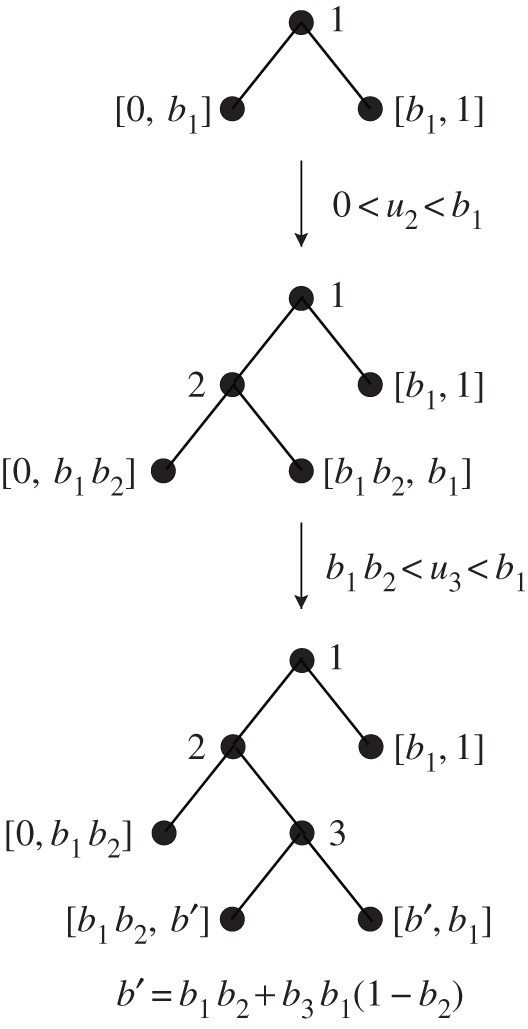
An example of construction for n=4.

In words, at each step *i* the labels of the leaves form a partition of the interval [0,1]. We find the next leaf to be split by checking which interval contains the corresponding $u_{i}$ and then $b_{i}$ is used to split the interval of that former leaf, say with length ℓ, into two intervals of lengths $b_{i}\ell$ and $(1-b_{i})\ell$. The internal node just created is then labelled by *i* to record the order of the splits. At the end of step *i*, the tree has i+1 leaves, and so we stop the procedure at step n−1. Figure 1 shows an example of such construction for n=4.

Note that once the realization of the generating sequence has been fixed, the creation of the ranked planar binary tree has no extra randomness. Below, we shall study the random tree obtained under the assumption that the generating sequence is a sequence of i.i.d. pairs ${\left(U_{i},B_{i}\right)}_{i\in N}$, where $U_{i}∼\text{Unif}[0,1]$ and $B_{i}∼B(\alpha +1,\beta +1)$.

### 2.3. Generating, organizing and deleting process

We can complete the organizing procedure to obtain an incremental construction of a tree with splitting (or reproduction) and freezing (or death) events. In this new process, freezing will correspond for example to a species becoming extinct (so that it cannot speciate later): such a leaf will be marked with a star and cannot be chosen to split in later steps. For this, we need to augment the generating sequence to include two more coordinates, which will decide whether the next step is a split or a freezing, and which leaf is frozen in the second case.

More precisely, let $\left(V_{1},V_{2},\ldots \right)$ and $\left(D_{1},D_{2},\ldots \right)$ be two independent sequences of i.i.d. random variables with a uniform distribution on [0,1] (independent of $\left(G_{1},G_{2},\ldots \right)$). Let also δ∈[0,1) be a fixed number corresponding to the probability that the next event is a freezing event and not a split. We augment the generating sequence into the following sequence of quadruples ${\left({\tilde{G}}_{i}=(U_{i},B_{i},V_{i},D_{i})\right)}_{i\in N}$.

Let now ${\left({\tilde{g}}_{i}=(u_{i},b_{i},v_{i},d_{i})\right)}_{i\in N}$ be a realization of the new generating sequence. Again, we start with a single root node, labelled by the interval [0,1] and proceed incrementally, until the tree created has *n* active (i.e. not frozen) leaves or no active leaves. At each step *i*, we decide to freeze if $v_{i}<\delta$ and split otherwise. If $v_{i}<\delta$, then we freeze the leaf node whose interval label contains $d_{i}$ by marking it with a star (if it was already marked, then nothing changes). If $v_{i}\geq \delta$, we find the leaf node whose interval label contains $u_{i}$ as before. If the corresponding leaf is still active, we split it according to the procedure described in the organizing map. If that leaf is frozen, then the event is cancelled. Alternatively, we can have a construction where the distribution is conditional over the currently active leaf intervals.

**Figure 2.**
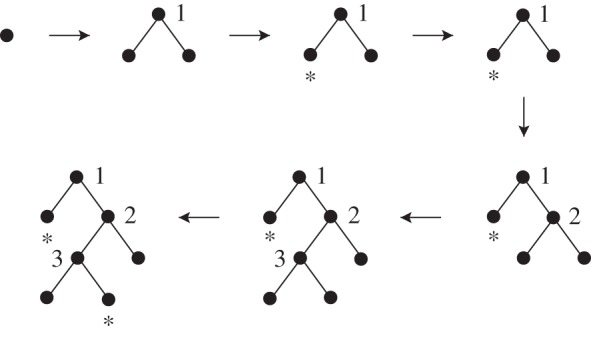
Example of a realization of the generating, organizing and deleting process. Here, we only record the labels of the internal nodes (the split ranking) and the stars indicating a frozen leaf, but each leaf is also labelled by an interval as in the organizing process. We start with a single node. During the first step, $v_{1}\geq \delta$ and so the node is split and becomes labelled by 1. Next, $v_{2}<\delta$ and $d_{2}$ belongs to the interval labelling the left leaf, so that this leaf becomes frozen. During the third step, whatever the value of $v_{3}$, the affected leaf chosen according to where $u_{i}$ or $d_{i}$ sits lies among the frozen leaves and so nothing happens. The next two steps are such that $v_{i}\geq \delta$ and the leaves chosen to split are both active. In the final step, $v_{6}<\delta$ and $d_{6}$ belongs to the interval labelling the right child leaf of node 3, which therefore becomes frozen.

Figure 2 gives an example of realization of the generating, organizing and deleting process. Of course, this procedure is particular in the sense that we may have chosen more general distributions for the variables $D_{i}$ dictating the choice of the leaf becoming frozen.

## 3. Properties of the Beta-splitting evolutionary trees

Keeping track of the generating sequence is useful to carry on the incremental construction and add new leaves to the tree. However, in most applications the object of interest is the (unlabelled) ranked planar binary tree obtained by keeping the labels of the internal nodes (giving the ranking of the splits) and by erasing the leaf nodes' interval labels whose widths give their speciation potentials that are yet to be observed. Thus, this is the random tree of interest in this section.

### 3.1. Probability of a given tree

All trees here are rooted and binary. First, let us give the probability of obtaining a given tree through the random generating and the non-random organizing processes.

For a given (unlabelled) ranked planar tree, and an internal node labelled by *i*, let us write $n_{i}^{L}$ (resp., $n_{i}^{R}$) for the number of internal nodes in the left (resp., right) subtree below node *i*. In particular, if node *i* subtends two leaves, then $n_{i}^{L}=0=n_{i}^{R}$.

Theorem 3.1 *For any unlabelled ranked planar binary tree τ with n leaves, we have*

3.1 $$\begin{array}{rl} P(\tau ) & =\prod_{i=1}^{n-1}\left\{\frac{1}{B(\alpha +1,\beta +1)}\int_{0}^{1}b_{i}^{n_{i}^{L}+\alpha }{\left(1-b_{i}\right)}^{n_{i}^{R}+\beta }db_{i}\right\}\\ & =\prod_{i=1}^{n-1}\frac{B(n_{i}^{L}+\alpha +1,n_{i}^{R}+\beta +1)}{B(\alpha +1,\beta +1)}, \end{array}$$

*where* B(α,β) *was defined in* (1.2).

**Proof outline.**

Remember that if a leaf is labelled by an interval [a,b], the probability that it is split during the *i*th step is b−a, the probability that the uniform random variable $U_{i}$ falls within [a,b]⊂[0,1]. If it is chosen to split, it is given label *i* and the left and right leaves created are labelled by intervals of respective lengths $B_{i}(b-a)$ and $\left(1-B_{i})(b-a\right)$. Then these intervals may split later, but into intervals of lengths that are always proportional to $B_{i}$ or $1-B_{i}$ (respectively). Now the probability of the tree *τ* is the product of the n−1 probabilities of choosing a given leaf to split at each step, each of which is equal to the length of the interval labelling that leaf. As a consequence, each split occurring in the left subtree below node *i* brings in another $B_{i}$ in the product, or another $1-B_{i}$ if the split occurs in the right subtree below node *i*. Averaging over the possible values of the $B_{i}$'s, which are independent B(α+1,β+1) random variables, yields the result. ▪

Remark 3.2 This construction is different from Aldous' interpretation in terms of splitting intervals that starts by uniformly scattering the given *n* leaf nodes as ‘particles’ on the unit interval and splitting the interval at a random point with density *f*. This splitting is repeated recursively on subintervals exactly as we do, i.e. splitting each interval [a,b] at a point a+X(b−a), where the *X*'s are independent with density *f*. Splitting stops when each subinterval contains only one leaf particle while splits that result in one of the intervals being empty (without any leaf particles in it) are not allowed. See the supplementary material of Blum & François [8] for a discussion on the relationship between Aldous' Beta-splitting model and this incremental construction.

### 3.2. Examples

In all the examples given below, we focus on the symmetric case α=β. Some of the formulae given below are easily generalized to the case α≠β.

The most important example is the case β=0, which corresponds to the Yule model of pure births that is used in many models of phylogenies.

Recall that B(α,β) is related to the Gamma function Γ by the equality

3.2 $$B(\alpha ,\beta )=\frac{\Gamma (\alpha )\Gamma (\beta )}{\Gamma (\alpha +\beta )},\;\alpha ,\beta >0,$$

and that Γ(β)=(β−1)!=(β−1)(β−2)⋯2⋅1 if β∈N. Using (3.1) with α=β=0, we have

3.3 $$P(\tau )=\prod_{i=1}^{n-1}\frac{n_{i}^{L}!n_{i}^{R}!}{(n_{i}^{L}+n_{i}^{R}+1)!}=\frac{1}{(n-1)!},$$

where the second equality is obtained by observing that $n_{i}^{L}+n_{i}^{R}+1$ is the number of internal nodes of the tree rooted at node *i*, which is the left or the right subtree below the mother node of *i*. Hence, each term $n_{i}^{L}!$ in the numerator of the product cancels with the term in the denominator that corresponds to the left child node of *i*, except if $n_{i}^{L}=0$ and the left child node of *i* is a leaf. But in this case, 0!=1 by convention. The same holds true for each of the $n_{i}^{R}!$. Likewise, the terms in the denominator which are not compensated by some term in the numerator are those corresponding to internal nodes having no mother nodes. But the only such node is the root (i=1), with $n_{1}^{L}+n_{1}^{R}+1=n-1$. This gives us the result.

Remark 3.3 This construction is very different from the standard evolutionary construction of the Yule tree, in which the next leaf to split is chosen uniformly at random among the current set of leaves. Here the choice of the next split is dictated by the lengths of the intervals labelling the current leaves, which will all be distinct with probability one. However, averaging over the law of the generating sequence (when α=β=0) yields the same distribution on ranked planar binary trees.

Using the above property of the Gamma function, we can also give explicit values for the probability of a tree when α=β is a non-negative integer: if β∈N∪{0}, then

3.4 $$P(\tau )=\prod_{i=1}^{n-1}\frac{(n_{i}^{L}+\beta )!(n_{i}^{R}+\beta )!(2\beta +1)!}{(n_{i}^{L}+n_{i}^{R}+2\beta +1)!{\left(\beta !\right)}^{2}}.$$

Thirdly, when *β* is a non-negative integer, we have

$$\Gamma \left(\beta +\frac{1}{2}\right)=\frac{(2\beta )!}{2^{2\beta }\beta !}\sqrt{\pi }.$$

As a consequence, another example in which the probability of a tree has an explicit form is the case where α=β=b−1/2, with b∈N∪{0}:

$$P(\tau )=\prod_{i=1}^{n-1}\frac{(2n_{i}^{L}+2b)!(2n_{i}^{R}+2b)!{\left(b!\right)}^{2}}{4^{n_{i}^{L}+n_{i}^{R}}(n_{i}^{L}+b)!(n_{i}^{R}+b)!(n_{i}^{L}+n_{i}^{R}+2b)!(2b)!}.$$

To the best of our knowledge, the cases α=β∈N and $\beta +\frac{1}{2}\in N\cup \{0\}$ correspond to no well-studied models of trees.

**Figure 3.**
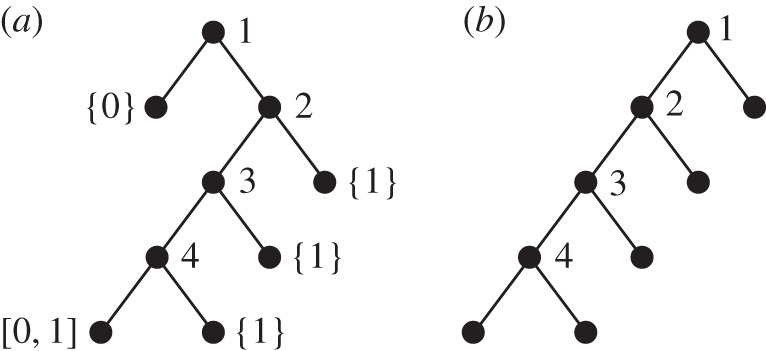
(*a*) An example of realization of a tree corresponding to the limiting case β=−1, and (*b*) the comb which is the only possible non-planar tree that can be generated in this case.

To see how the global shape of the tree (and in particular its balance) evolves as *β* goes from −1 to +∞, let us consider the two extreme cases. The corresponding processes cannot be defined directly as −1 and +∞ lie out of the range of the possible *β*'s, but we can capture the essence of the resulting (random) tree by taking limits in *β*. First, as β→−1, the B(β+1,β+1) distribution gives more and more weight to the boundaries 0 and 1. In the limit, the random variables $B_{i}$ should then take the values 0 or 1, each with probability $\frac{1}{2}$. In this case, the root is first split into a leaf with label [0,1] and another leaf with label {0} or {1} (i.e. an interval reduced to a single point). The leaf that receives the label [0,1] is the left one with probability $\frac{1}{2}$. Next, the uniform random variable $U_{2}$ belongs to the interval [0,1] with probability one, so that the leaf labelled by [0,1] is necessarily that chosen to split. Again, it is split into two leaves with labels [0,1] and {0} or {1}, implying that the next leaf to split is that inheriting the full interval [0,1] with probability one. The reasoning can be carried on until step n−1. Hence, morally the tree corresponding to α=β=−1 is a fully unbalanced tree, with a single backbone from which the *n* leaves are hanging. The backbone is extended at each step by choosing one of the two leaves created in the previous step, each with probability $\frac{1}{2}$. See figure 3 for an example with n=5.

Let us now consider the limit β→+∞. Using (3.4) and the fact that $(b+i)!/b!∼b^{i}$ as b→∞ (meaning that the ratio of both terms tends to 1), we can pass to the limit β→∞ and obtain that

$$\lim_{\beta \to +\infty }P(\tau )=\prod_{i=1}^{n-1}\frac{1}{2^{n_{i}^{L}+n_{i}^{R}}}.$$

Because in the balanced trees the internal nodes below a given node are equally split between the left and right subtrees hanging from that node, the sum $n_{i}^{L}+n_{i}^{R}$ decreases with *i* faster than in more unbalanced trees. This means that for very large *β*'s, approximately balanced trees will have much higher probabilities than unbalanced ones. For instance, any of the fully unbalanced trees $\tau_{u}$ will have probability

$$P(\tau_{u})=1/[2^{n-2}\cdot 2^{n-3}\cdots 2\cdot 1]=2^{-(n-1)(n-2)/2}.$$

On the other hand, if $n=2^{N}$ is a power of 2, the probability of any of the fully balanced trees $\tau_{b}$ is equal to

$$\begin{array}{rl} P(\tau_{b}) & =1/[2^{n-2}.{\left(2^{n/2-2}\right)}^{2}\cdot {\left(2^{n/4-2}\right)}^{4}\cdots {\left(2^{2}\right)}^{2^{N-2}}\cdot {\left(2^{0}\right)}^{2^{N-1}}]\\ & =\prod_{k=0}^{N-1}{\left(2^{n/2^{k}-2}\right)}^{-2^{k}}=\prod_{k=0}^{N-1}2^{2^{k+1}-n}=2^{-n(N-2)-2}. \end{array}$$

Indeed, any subtree pending from a node at level k∈{0,…,N−1} (level 0 being that of the root, level $N=\log_{2}(n)$ that of all the leaves) has $n/2^{k}$ leaves, and so $(n/2^{k})-2$ internal nodes below its root. Furthermore, there are $2^{k}$ internal nodes at level *k*. Together with (3.1), this gives us the result.

Using the results obtained in the next section, we can further compute the probability of producing a fully unbalanced (unranked non-planar) tree as being equal to

$$P(t_{u})=2^{n-2}\;P(\tau_{u})=\left\{\begin{array}{ll} 1 & \text{if }\beta =-1,\\ {}[6pt]\frac{2^{n-2}}{(n-1)!} & \text{if }\beta =0,\\ {}[8pt]2^{-(n-2)(n-3)/2} & \text{if }\beta =+\infty . \end{array}\right.$$

Likewise, the probability of producing a fully balanced tree with $n=2^{N}$ tips is given by

$$P(t_{b})=\frac{(n-1)!}{\prod_{k=0}^{N-1}{\left(n/2^{k}-1\right)}^{2^{k}}}P(\tau_{b}).$$

Table 1 gives a few examples of these probabilities for different values of $n=2^{N}$ and β=−1,0,+∞.

**Table 1.** Probability of sampling a comb tree or a fully balanced tree for different values of *n* and *β*. As explained in the text, larger values of *β* correspond to higher probabilities of sampling a balanced tree.

	*n*	4	8	32	1024
β=−1	comb	1	1	1	1
	balanced	0	0	0	0
β=0	comb	0.667	$1.27\times 10^{-2}$	$1.31\times 10^{-25}$	$8.49\times 10^{-2330}$
	balanced	0.333	$1.59\times 10^{-2}$	$9.10\times 10^{-12}$	$1.04\times 10^{-417}$
β=+∞	comb	0.5	$3.05\times 10^{-5}$	$1.13\times 10^{-131}$	$2.09\times 10^{-157057}$
	balanced	0.5	$7.81\times 10^{-2}$	$2.36\times 10^{-7}$	$1.26\times 10^{-247}$

Finally, we have shown that the family of Beta-splitting trees defined in Blum & François [8] and generalized in this article includes a one-parameter family containing the classical Yule (ranked planar) tree. For small *β*'s (close to −1), the corresponding trees are unbalanced with high probability, whereas for large *β*'s the tree distribution is concentrated on balanced trees. The family of Beta-splitting trees indexed by α,β>−1 thus covers a very wide range of possible topologies.

## 4. Other tree resolutions

Recall that a tree in this paper is always rooted and binary. Up to now we have focused on ranked planar trees with *n* leaves that keep records of the order in which splits occur and give an asymmetric role to the left and right child nodes of an internal node. These (n−1)! many trees are in bijective correspondence with permutations of {1,…,n−1} through the *increasing binary tree-lifting* operation (see [18, Ex. 17, p. 132]). However, we may be interested in coarser resolutions of the trees generated by our Beta-splitting procedure, especially those resolutions of interest to evolutionary biologists. To the best of our knowledge, explicit formulae for the probability of any tree at these resolutions are not available in the literature as a function of *α* and *β* (even for the symmetric case when α=β). This is because the cardinality of the inverse image from a fine to a coarser tree resolution needs to be computed for any tree in the coarser resolution. Such probabilities can be directly useful in simulation-intensive inference.

### 4.1. Probability of unranked planar trees

Here, we keep the lack of symmetry between the child nodes, but do not record the order of the splits. As explained in the Introduction, this may be of interest, for example, if we assume that there is a lack of symmetry between the two species created during a speciation event, say due to one species being the ancestor and other being the descendant, but we do not want to reconstruct the temporal order in which the speciation events occurred. In the context of transmission trees, we may only be interested in the infector–infectee relation for each transmission event and not in the ranking of transmission events given by their relative temporal order.

Since we do not label the internal nodes, let I(t) denote the set of all internal nodes of a planar tree t and let us extend the notation $n_{i}^{L}$ and $n_{i}^{R}$, i∈I, for the number of internal nodes in the left and right subtrees below node *i* to this unlabelled case. The probability of obtaining a given (unranked) planar binary tree t through the Beta-splitting generating and organizing processes is given by the following lemma.

Lemma 4.1 *Let* t *be a planar binary tree. We have*

P(t)=∏i∈I(t)niL+niRniL∏i∈I(t)B(niL+α+1,niR+β+1)B(α+1,β+1)=(n−1)!∏i∈I(t)B(niL+α+1,niR+β+1)(niL+niR+1)B(α+1,β+1).

Indeed, recall that the second product in the right-hand side of the first equality above is the probability of a given ranked planar tree corresponding to the unranked tree t. Since it does not depend on the ranking, there remains to count the number of ranked trees whose unranking gives t. Now, to rank the internal nodes of t, at each split we have to decide which of the remaining integer labels go to the left or to the right subtree below the corresponding node. This gives us $\text{Binomial}(n_{i}^{L}+n_{i}^{R},n_{i}^{L})$ choices, hence the first product term in P(t). This product of binomial coefficients is called the shape functional [19], the Catalan coefficient [20] and is the solution to an enumerative combinatorial exercise [21, ch. 3, Ex. 1.b, p. 312].

As in the case α=β=0 (see the derivation of (3.3)), the simplification leading to the last equality comes from the fact that $n_{i}^{L}+n_{i}^{R}+1$ is the number of internal nodes of the subtree rooted at node *i*, so that most factorial terms cancel out in the product over I(t).

### 4.2. Probability of ranked non-planar trees

In this case, we keep the ranking but give a symmetric role to the left and right child nodes of an internal node. These trees are termed *evolutionary relationships* by Tajima [22], who shows that there are $2^{n-1-c(t)}$ ranked planar trees for a given ranked non-planar tree t, where c(t) is the number of cherry nodes, i.e. sub-terminal nodes with two child nodes. For a quick justification of Tajima's result, suppose we want to turn the ranked non-planar tree t into a planar tree. For each of the n−1 internal nodes of t, there are two choices for the child node that is said to be ‘left’ except if they are both leaves (i.e. the internal node is a cherry node). Indeed, in this case they carry no ranking that would make them distinguishable.

Since the probability of a ranked non-planar tree does not depend on the planarity, provided α=β, the probability of t is simply the product of the probability of a corresponding ranked planar tree, times the number of ranked planar trees corresponding to t. That is,

$$P(t)=2^{n-1-c(t)}\prod_{i=1}^{n-1}\frac{B(n_{i}^{L}+\beta +1,n_{i}^{R}+\beta +1)}{B(\beta +1,\beta +1)}.$$

Note that when α≠β, we need to sum over all $2^{n-1-c(t)}$ ranked planar trees that map to the ranked non-planar tree t (since in this case $B(n_{i}^{L}+\alpha +1,n_{i}^{R}+\beta +1)\neq B(n_{i}^{R}+\alpha +1,n_{i}^{L}+\beta +1)$), and this may not be computationally feasible for large *n*.

### 4.3. Probability of trees

This is the case of (rooted binary) unranked non-planar trees or simply trees (also called phylogenetic tree shapes). There are $2^{n-1-s(t)}$ unranked planar trees that correspond to a tree *t*, where s(t) is the number of internal nodes of *t* that have isomorphic left and right subtrees. See Sainudiin *et al.* [23] for a proof by induction. And all these unranked planar trees that correspond to *t* have the same probability provided α=β. One can intuitively understand this by noting that there are two unranked planar embeddings for each internal node of *t* that does not have isomorphic subtrees on its left and right descendant nodes. Thus, the probability of a tree *t* if α=β is

$$P(t)=2^{n-1-s(t)}(n-1)!\prod_{i\in I(t)}\frac{B(n_{i}^{L}+\beta +1,n_{i}^{R}+\beta +1)}{\left(n_{i}^{L}+n_{i}^{R}+1)B(\beta +1,\beta +1\right)}.$$

Once again if α≠β we need to sum over all $2^{n-1-s(t)}$ unranked planar trees that map to the (unranked non-planar) tree *t* and this may not be computationally feasible for large *n*.

## 5. Continuous-time process

Up to now we have described the generating–organizing–deleting process in discrete time over ranked planar trees and given the probabilities over various equivalence classes of trees. However, one may want to formulate a continuous-time version of this process in order to have a more precise description of the evolutionary relationships including how much time elapsed between two speciation or extinction events.

To do so, recall that we fix two parameters α,β>−1 characterizing the way in which leaf intervals are split, and the probability δ∈[0,1) of a freezing during the next event (if δ=0, only splits occur). Let us also fix a rate λ>0 of events. One way to formulate a continuous-time generating–organizing–deleting process is the following: suppose the current interval length of the *j*th active leaf is $L_{j}$. Then each active leaf *j* splits at rate $\lambda (1-\delta )L_{j}$ or becomes frozen at rate $\lambda \delta L_{j}$. When a split occurs, the internal node created during the event is labelled by the first integer *N* larger than all integer labels in the current tree, and the two leaves created are labelled by intervals that are obtained by splitting the (former) interval label of node *N* using $b_{N}$ (that is, if that interval is [a,b], the new leaves are labelled by $\left[a,a+b_{N}(b-a)\right]$ and $\left[a+b_{N}(b-a),b\right]$ as before).

Lemma 5.1 *The discrete tree embedded in the continuous-time planar ranked tree stopped after the nth event has the same law as the* (*ranked planar*) *tree obtained from the generating, organizing and deleting process stopped after the nth effective event. By effective event, we mean an event affecting an active leaf and therefore leading to a change in the tree*.

Proof. Let us write $L_{\text{act}}$ for the sum over all active leaves of their interval lengths $L_{j}$, and $I_{\text{act}}$ for the union of the corresponding intervals. Hence, $1-L_{\text{act}}$ is the length of the set $[0,1]\setminus I_{\text{act}}$ corresponding to all frozen leaves. Let us check for both random trees that, conditionally on the current state of the process:

(i) The next (effective) event is a split with probability 1−δ or a freezing with probability *δ*.
(ii) If it is a split, then the probability that leaf *j* is chosen to split is $L_{j}/L_{\text{act}}$.
(iii) If it is a freezing, then the probability that leaf *j* is chosen to freeze is also $L_{j}/L_{\text{act}}$.

The result is an easy consequence of the construction of continuous-time jump processes for the tree embedded in the continuous-time procedure (in essence, if we have a countable collection of events such that event *i* happens at rate $\mu_{i}$, then the first event to occur is the *j*th one with probability $\mu_{j}/\sum_{i}\mu_{i}$).

For the tree constructed from the discrete process ‘restricted’ to the effective events, observe first that the coordinates $\left\{u_{i},\;d_{i},\;i\in N\right\}$ of the organizing process are recorded in the ranked planar tree only through the choices of the next leaf to be affected. Also, the coordinates $\left\{v_{i},\;i\in N\right\}$ appear only through the types of the next events to occur. As a consequence, the law of the tree emanating from this construction depends only on the probabilities that each of these quantities belongs to a given set, conditionally on the fact that the leaf chosen is active. That is, the probability that the next effective event is a freezing is

$$P(V_{i}<\delta \;|\;D_{i}\in I_{\text{act}})=\frac{P(V_{i}<\delta )P(D_{i}\in I_{\text{act}})}{P(D_{i}\in I_{\text{act}})}=\delta$$

since $V_{i}$ and $D_{i}$ are independent. Likewise, the probability of the next effective event being a split is 1−δ, which proves (i). Next, conditionally on the next event being a split, the probability that leaf *j* is chosen is (again by the independence of $U_{i}$ and $V_{i}$)

$$P(U_{i}\in I_{j}\;|\;V_{i}>\delta ,\;U_{i}\in I_{\text{act}})=P(U_{i}\in I_{j}\;|\;U_{i}\in I_{\text{act}})=\frac{L_{j}}{L_{\text{act}}}.$$

This proves (ii), and (iii) can be obtained in the same way. Points (i)–(iii) then enable us to conclude that the topologies and rankings of both trees have the same law.

There remains to show that, conditionally on the topology and ranking, the interval labels of the leaves are identical in distribution. Note that they are not *a priori* identical with probability one since the continuous-time construction uses the variables $(B_{1},B_{2},\ldots ,B_{n}),$ whereas the discrete-time construction uses the variables $\left(B_{i_{1}},B_{i_{2}},\ldots ,B_{i_{n}}\right)$, where $i_{1}<i_{2}<\cdots <i_{n}$ are the random indices of the effective events. But the event that $i_{j}=k$ depends only on the generating sequence ${\left({\tilde{G}}_{i}\right)}_{1\leq i\leq k-1}$, since it depends only on the current length of all active leaves. Consequently, $i_{j}$ and $B_{i_{j}}$ are independent random variables (recall that all components of $\left({\tilde{G}}_{i}\right)$ are independent of each other). A simple argument then shows that the law of $\left(B_{i_{1}},B_{i_{2}},\ldots ,B_{i_{n}}\right)$ is the same as the law of $\left(B_{1},B_{2},\ldots ,B_{n}\right)$, which in turn guarantees that the interval labels of the leaves are also equal in law. Lemma 5.1 is proved. ▪

## Supplementary Material

Supplementary material : Algorithm related to the construction in the main paper, an example and an additional mathematical property of the model

## Data accessibility

The code developed for this work is given in the electronic supplementary material. It is also publicly shared at https://cloud.sagemath.com/projects/2c5f7f68-e689-4c70-a4b4-5b5d4dc4f93f/files/2015-10-27-082849.sagews.
