# Supplementary material for: A Beta-splitting model for evolutionary trees
Source: R Soc Open Sci. 2016 May 11;3(5):160016. doi: 10.1098/rsos.160016 (PMC4892442; doi:10.1098/rsos.160016)
Supplement: Supplementary material : Algorithm related to the construction in the main paper, an example and an additional mathematical property of the model [file rsos160016supp1.pdf]

A Beta-splitting Model for Evolutionary Trees
Supplementary Material

Raazesh Sainudiin and Amandine Véber

Algorithm

This code is publicly shared at `https://cloud.sagemath.com/projects/2c5f7f68-e689-4c70-a4b4-5b5d4dc4f93f/files/2015-10-27-082849.sagews`. The code was mainly used to aid intuition during this study and is not written to be efficient for large scale simulation studies. The core Algorithms for the generating and organizing processes are presented as SageMath/python code instead of pseudo-code in order to communicate the Algorithms used in this study in a more concrete and reproducible manner. This also allows the reader to perform computational experiments in SageMath/python immediately to further extend this work.

The function `split01ScaledCD` takes the interval I and splits it into 2 intervals of lengths $x|I|$ and $(1-x)|I|$ (with $x \in [0,1]$).

```
def split01ScaledCD(x,I):
    '''x \in [0,1], c=I[0] < d=I[1]'''
    c=I[0]; d=I[1];
    return [[c,c+(d-c)*x],[c+(d-c)*x,d]];
```

The function `SplittingPermutation` translates a sequence of n real numbers (our splitting points, later) into a permutation of $[n] = \{1, \dots, n\}$ by returning a list of n integers such that the i-th element of the list is the index of the i-th smallest number in the initial sequence. For example, `SplittingPermutation`$([1.1, 10, -1, 2.5])=[3, 1, 4, 2]$.

```
def SplittingPermutation(splitpointsequence):
    '''return the permutation of [n] given by the map from
    the sequence of n real numbers in the list
    splitpointsequence to an ordering by indices in [n]'''
    sss=sorted(splitpointsequence)
    return tuple([splitpointsequence.index(i)+1 for i in sss])
```

In the function `MakePartitionAndTree`, we construct m samples of a tree with n splits (or $n+1$ leaves), using the $\mathcal{B}(a+1, b+1)$ distribution of the coordinates B_i of the generating sequence. We first obtain the sequence of points in $[0,1]$ where the splits occur according to the generating sequence and store them in `SplitPoints`. Then we use the standard `binary_search_insert` method to obtain a binary search tree that organizes the points in `SplitPoints` into an unranked planar binary tree. Recall that the split points are inserted from the root of the tree such that the new point that is smaller/larger than the point at the root node descends into the left/right subtree of the root and recursively takes left/right subtree depending on whether it is lesser or greater than the next point it encounters at an internal node that is already stored in the tree. Each of these trees is recorded at several resolutions: the ranked planar trees (recorded in the list `SplittingPermutationSamples`, unranked planar trees (recorded in `PlanarShapeSamples`) and unranked non-planar trees (recorded in `PhyloShapeSamples`).

For the finest resolution of ranked planar trees, we use the bijection between ranked planar trees with $n+1$ leaves and permutations of $[n]$ (note that the cardinality of each set is $n!$). This bijection is

detailed in Flajolet and Sedgewick (2009), Ex. 17, p. 132, and follows this idea. Say the permutation of $[n]$ of which we want to draw the tree is $[i_1, i_2, \dots, i_n]$. We first construct the planar skeleton of the ranked internal nodes incrementally by starting with a single node. To place the second node, check whether $i_2 < i_1$, in which case the left child of the root becomes node 2, or whether $i_2 > i_1$ and the right child node of the root becomes node 2. To place the third node, find whether it goes to the left or to the right of the root by checking whether $i_3 < i_1$ or $i_3 > i_1$. Once this is decided, if the second and third nodes are on the same side of the root, then compare i_3 to i_2 to decide whether node 3 should be the left or right child of node 2. Proceeding in the same way for the other terms of the permutation, we construct a planar ranked skeleton with n nodes. There remains to attach the $n+1$ leaves to the terminal nodes of the skeleton to obtain a ranked planar tree with n splits. For ease of representation, the output corresponding to the ranked planar trees is thus the list `SplittingPermutationSamples` recording the permutations corresponding to the m trees.

```
def MakePartitionAndTree(n,m,a,b):
    '''This creates m independent trees with n+1 leaves and alpha=a, beta=b
       n >= 1, where n+1 is the number of leaves
       m is the number of replicates
       a,b>-1, where (a+1,b+1) are the parameters of the beta distribution'''
    PlanarShapeSamples=[];
    PhyloShapeSamples=[];
    SplittingPermutationSamples=[];
    BetaD = RealDistribution('beta', [a+1, b+1],seed=0)
    show(BetaD.plot(xmin=0,xmax=1),figsize=[7,2]);
    print '\n';
    for reps in range(m):
        # generating i.i.d. samples from BetaD
        B=[BetaD.get_random_element() for _ in range(n)]
        #initialize
        SplitPoints=[B[0]]# keep order of split points
        Splits=split01ScaledCD(B[0],[0,1])

        #iterate
        for i in range(1,n):
            Widths=[x[1]-x[0] for x in Splits];
            W = GeneralDiscreteDistribution(Widths);
            nextSplitI=Splits[W.get_random_element()];
            Splits.remove(nextSplitI);
            NewLeaves=split01ScaledCD(B[i],[nextSplitI[0], nextSplitI[1]]);
            # find the split point between the new leaves
            RescaledG=NewLeaves[0][1];
            SplitPoints.append(RescaledG);
            Splits.append(NewLeaves[0]);
            Splits.append(NewLeaves[1]);
        SplittingPermutationSamples.append(SplittingPermutation(SplitPoints));
        # insert the split points into the tree
        t = LabelledBinaryTree(None)
        for i in range(0,n):
            t = t.binary_search_insert(SplitPoints[i]);
        sh=t.shape();
        PlanarShapeSamples.append(sh);
```

```
        PhyloShapeSamples.append(Graph(sh.to_undirected_graph(with_leaves=True),
                                                         immutable=True));
    return (PlanarShapeSamples,PhyloShapeSamples,SplittingPermutationSamples);
```

We also provide some additional functions computing the probabilities of a given tree at a particular resolution under the Beta-splitting model.

```
def splitsSequence(T):
    '''return a list of tuples (left,right) split sizes at each split node'''
    l = []
    T.post_order_traversal(lambda node:
       l.append((node[0].node_number(),node[1].node_number())))
    return l

def isIso(N):
    '''does node N of binary tree have the same left and right subtree shapes
    (are left and right subtrees of node N in tree isomorphic)'''
    L=Graph(N[0].canonical_labelling().shape().to_undirected_graph(with_leaves=True),
                                              immutable=True)
    R=Graph(N[1].canonical_labelling().shape().to_undirected_graph(with_leaves=True),
                                              immutable=True)
    return 1 if L==R else 0

def numIso(T):
    '''number of internal nodes that have isomorphic left and right sub-trees'''
    l = []
    T.post_order_traversal(lambda node:l.append(isIso(node)))
    return sum(l)

def prob_RPT(T,a,b):
    '''probability of ranked planar tree T under beta-splitting model
       a,b>-1, where (a+1,b+1) are the parameters of the beta distribution'''
    # non-cherry splits
    ncspS=filter(lambda x: x!=(0,0), splitsSequence(T))
    return prod(map(lambda x:beta(x[0]+a+1,x[1]+b+1)/beta(a+1,b+1),ncspS))

def prob_PT(T,a,b):
    '''probability of planar tree T under beta-splitting model
       a,b>-1, where (a+1,b+1) are the parameters of the beta distribution'''
    # non-cherry splits
    ncspS=filter(lambda x: x!=(0,0), splitsSequence(T))
    return prod(map(lambda x: binomial(x[0]+x[1],x[1])*
            beta(x[0]+a+1,x[1]+b+1)/beta(a+1,b+1),ncspS))

def prob_RT(T,a,b):
    '''probability of ranked (nonplar) tree T under beta-splitting model
       a,b>-1, where (a+1,b+1) are the parameters of the beta distribution'''
    assert(a==b)
    spS=splitsSequence(T)
    numSplits=len(spS)
```

```
    # non-cherry splits
    ncspS=filter(lambda x: x!=(0,0), spS)
    numCherries=numSplits-len(ncspS)
    probRPT = prod(map(lambda x:beta(x[0]+a+1,x[1]+b+1)/beta(a+1,b+1), ncspS))
    return 2^(numSplits-numCherries)*probRPT

def prob_T(T,a,b):
    '''probability of tree T (phylo tree shape) under beta-splitting model
       a,b>-1, where (a+1,b+1) are the parameters of the beta distribution'''
    assert(a==b)
    spS=splitsSequence(T)
    numSplits=len(spS)
    # non-cherry splits
    ncspS=filter(lambda x: x!=(0,0), spS)
    probPT = prod(map(lambda x: binomial(x[0]+x[1],x[1])*
              beta(x[0]+a+1,x[1]+b+1)/beta(a+1,b+1),ncspS))
    numIsoSplits=numIso(T)
    return 2^(numSplits-numIsoSplits)*probPT

def stats_probs_Tree(T,a,b):
    '''probability of various resolutions of tree T under beta-splitting model
       a,b>-1, where (a+1,b+1) are the parameters of the beta distribution'''
    spS=splitsSequence(T)
    numSplits=len(spS)
    # non-cherry splits
    ncspS=filter(lambda x: x!=(0,0), spS)
    numCherries=numSplits-len(ncspS)
    probRPT = prod(map(lambda x:beta(x[0]+a+1,x[1]+b+1)/beta(a+1,b+1), ncspS))
    catCoeff = prod(map(lambda x:binomial(x[0]+x[1],x[1]),ncspS))
    # prob of (non-ranked) planar tree
    probPT = catCoeff * probRPT
    probRT = 2^(numSplits-numCherries)*probRPT
    numIsoSplits=numIso(T)
    probT=2^(numSplits-numIsoSplits)*probPT
    return (numSplits,numIsoSplits,numCherries,catCoeff,probRPT,probPT,probRT,probT)
```

Example of Yule trees with 4 leaves

Here is a demonstration of the algorithm for the case of the Yule tree, $\alpha = \beta = 0$, with 4 leaves.

```
a=0; b=0; m=10000;
(bts,pts,sps)=MakePartitionAndTree(3,m,a,b)

def CountsDictWithFirstIndex(X):
    '''convert a list X into a Dictionary of counts or
    frequencies with first index of each Key saved'''
    CD = {}
    for i in range(len(X)):
        x=X[i]
```

```
        if (x in CD):
            CD[x][1] = CD[x][1]+1
        else:
            CD[x] = [i,1]
    return CD
```

`sps` gives the list of the $m = 10000$ ranked planar trees sampled by `MakePartitionAndTree`. The following function gives, for each of the trees encountered in `sps`, the theoretical probability of the tree under the Beta-splitting model with $a = b = 0$ and its empirical probability (i.e., its frequency in `sps`).

```
BtcCnts=CountsDictWithFirstIndex(sps)
for x in BtcCnts:
    print (sps[BtcCnts[x][0]],prob_RPT(bts[BtcCnts[x][0]],a,b).N(digits=4),
            (BtcCnts[x][1]/m).N(digits=4))

((1, 3, 2), 0.1667, 0.1700)
((3, 2, 1), 0.1667, 0.1666)
((2, 1, 3), 0.1667, 0.1625)
((3, 1, 2), 0.1667, 0.1664)
((1, 2, 3), 0.1667, 0.1683)
((2, 3, 1), 0.1667, 0.1662)
```

`bts` lists the 10000 unranked planar trees corresponding to the ranked planar trees in `sps`. The following function gives, for each of these trees, their theoretical and empirical probabilities.

```
BtcCnts=CountsDictWithFirstIndex(bts)
for x in BtcCnts:
    print (bts[BtcCnts[x][0]],
           prob_PT(bts[BtcCnts[x][0]],a,b).N(digits=5),(BtcCnts[x][1]/m).N(digits=5))

([., [[., .], .]], 0.16667, 0.17000)
([., [., [., .]]], 0.16667, 0.16830)
([[[., .], .], .], 0.16667, 0.16660)
([[., [., .]], .], 0.16667, 0.16620)
([[., .], [., .]], 0.33333, 0.32890)
```

More examples can be found at `https://cloud.sagemath.com/projects/2c5f7f68-e689-4c70-a4b4-5b5d4dc4f93f/files/2015-10-27-082849.sagews`.

A reversal property

Although Aldous' leaf deletion property does not seem to hold in general for the random tree obtained through the generating and organizing process, at the resolution of the unranked planar trees it is possible to define a transition kernel $\overleftarrow{\mathbb{P}}$ from the set of trees with $n+1$ leaves to the set of trees with n leaves in such a way that the tree obtained after (i) creating a tree with $n+1$ leaves thanks to the generating and organizing process, and (ii) choosing a (cherry) node to withdraw in order to come back to a tree with n leaves, has the same distribution as the tree obtained from running the generating and organizing process for only $n-1$ steps. That is, writing $\mathbf{T}_n$ for the random unranked planar tree with n leaves, we have for every tree $\mathbf{t}_n$ with n leaves:

$$\mathbb{P}(\mathbf{T}_n = \mathbf{t}_n) = \sum_{\mathbf{t}_{n+1}} \mathbb{P}(\mathbf{T}_{n+1} = \mathbf{t}_{n+1}) \overleftarrow{\mathbb{P}}(\mathbf{t}_{n+1} \to \mathbf{t}_n). \tag{1}$$

Indeed, let us set

$$\overleftarrow{\mathbb{P}}(\mathbf{t}_{n+1} \to \mathbf{t}_n) = \mathbb{P}(\mathbf{T}_n = \mathbf{t}_n \,|\, \mathbf{T}_{n+1} = \mathbf{t}_{n+1}). \tag{2}$$

Note that this probability is 0 if $\mathbf{t}_n$ and $\mathbf{t}_{n+1}$ are not compatible, that is if we cannot obtain $\mathbf{t}_{n+1}$ from $\mathbf{t}_n$ by splitting one of the leaves of $\mathbf{t}_n$. Then, we trivially have

$$\begin{aligned}\sum_{\mathbf{t}_{n+1}} \mathbb{P}(\mathbf{T}_{n+1} = \mathbf{t}_{n+1}) \overleftarrow{\mathbb{P}}(\mathbf{t}_{n+1} \to \mathbf{t}_n) &= \sum_{\mathbf{t}_{n+1}} \mathbb{P}(\mathbf{T}_{n+1} = \mathbf{t}_{n+1}) \mathbb{P}(\mathbf{T}_n = \mathbf{t}_n \,|\, \mathbf{T}_{n+1} = \mathbf{t}_{n+1}) \\ &= \mathbb{P}(\mathbf{T}_n = \mathbf{t}_n),\end{aligned}$$

which shows that (1) is satisfied.

Let us now give an explicit formula for the r.h.s. of (2) in the case where $\mathbf{t}_n$ and $\mathbf{t}_{n+1}$ are compatible. It is easier to come back to the resolution of ranked planar trees to compute the conditional probability appearing in the r.h.s. Indeed, as explained in the section on unranked planar trees, the probability of a given ranked planar tree τ_n does not depend on the ranking. As a consequence, conditionally on $\mathbf{T_n} = \mathbf{t}_n$, all ranked planar trees whose unranking yields $\mathbf{t}_n$ have the same probability $1/\#\mathbf{t}_n$ to be that created by the generating and organizing process, where $\#\mathbf{t}_n$ denotes the number of ranked trees corresponding to the unranked tree $\mathbf{t}_n$. Recall from the section on unranked planar trees that

$$\#\mathbf{t}_n = \prod_{i \in \mathcal{I}(\mathbf{t}_n)} \binom{n_i^L + n_i^R}{n_i^L}.$$

Writing $\mathcal{T}_n$ for the random ranked planar tree with n leaves and $\tau_n \prec \mathbf{t}_n$ to denote the fact that forgetting the ranking in the ranked planar tree τ_n yields $\mathbf{t}_n$, we have

$$\begin{aligned}\mathbb{P}(\mathbf{T}_n = \mathbf{t}_n \,|\, \mathbf{T}_{n+1} = \mathbf{t}_{n+1}) &= \sum_{\tau_{n+1} \prec \mathbf{t}_{n+1}} \mathbb{P}(\mathbf{T}_n = \mathbf{t}_n \,|\, \mathcal{T}_{n+1} = \tau_{n+1}) \mathbb{P}(\mathcal{T}_{n+1} = \tau_{n+1} \,|\, \mathbf{T}_{n+1} = \mathbf{t}_{n+1}) \\ &= \frac{1}{\#\mathbf{t}_{n+1}} \sum_{\tau_{n+1} \prec \mathbf{t}_{n+1}} \mathbb{P}(\mathbf{T}_n = \mathbf{t}_n \,|\, \mathcal{T}_{n+1} = \tau_{n+1}) \\ &= \frac{1}{\#\mathbf{t}_{n+1}} \sum_{\tau_{n+1} \prec \mathbf{t}_{n+1}} \sum_{\tau_n \prec \mathbf{t}_n} \mathbb{P}(\mathcal{T}_n = \tau_n \,|\, \mathcal{T}_{n+1} = \tau_{n+1}). \end{aligned} \tag{3}$$

Since we now work with ranked planar trees, for any tree τ_{n+1} with $n+1$ leaves the probability in the r.h.s. of (3) is zero unless τ_n is the tree τ_{n+1}^{-1} obtained by withdrawing the n-th split in τ_{n+1} (in which case the probability is 1). Hence, the r.h.s. in (3) can be written

$$\frac{1}{\#\mathbf{t}_{n+1}} \#\{\tau_{n+1} : \tau_{n+1} \prec \mathbf{t}_{n+1},\, \tau_{n+1}^{-1} \prec \mathbf{t}_n\}.$$

But now recall that $\mathbf{t}_n$ and $\mathbf{t}_{n+1}$ are assumed to be compatible. Hence, for every tree τ_n satisfying $\tau_n \prec \mathbf{t}_n$ there is one and only one way to add a last step to obtain a tree $\tau_{n+1} \prec \mathbf{t}_{n+1}$ (namely, add the missing split in the tree and label it by n). As a consequence, we obtain that

$$\mathbb{P}(\mathbf{T}_n = \mathbf{t}_n \,|\, \mathbf{T}_{n+1} = \mathbf{t}_{n+1}) = \frac{\#\mathbf{t}_n}{\#\mathbf{t}_{n+1}}. \tag{4}$$

Note that the same definition (2) would work at the resolution of the unranked non-planar trees, but finding an explicit expression for the quantity in the r.h.s. is difficult due to the many symmetries of non-planar trees.
